# Molecular Analysis of *Human Metapneumovirus* Detected in Patients with Lower Respiratory Tract Infection in Upper Egypt

**DOI:** 10.1155/2014/290793

**Published:** 2014-01-30

**Authors:** Mona S. Embarek Mohamed, Janine Reiche, Sonja Jacobsen, Amany G. Thabit, Mohamed S. Badary, Wolfram Brune, Brunhilde Schweiger, Ahmed H. Osmann

**Affiliations:** ^1^Department of Microbiology and Immunology, Faculty of Medicine, Assiut University, Assiut 71516, Egypt; ^2^National Influenza Center, Robert Koch Institute, 13353 Berlin, Germany; ^3^Heinrich Pette Institute, Leibniz Institute for Experimental Virology, 20251 Hamburg, Germany; ^4^Department of Chest Diseases, Faculty of Medicine, Assiut University, Assiut 71516, Egypt

## Abstract

*Introduction*. Since 2001, when *Human metapneumovirus* (HMPV) was isolated in the Netherlands, the virus has been detected in several continents. Although reports have confirmed the prevalence of HMPV worldwide, data from Egypt remain limited. HMPV plays an important role in respiratory tract infections in individuals of all ages particularly in children. This study was aimed at estimating the prevalence of HMPV in patients with community-acquired lower respiratory infection in Upper Egypt and characterizing the circulating Egyptian HMPV strains for the first time. *Materials and Methods*. From 2005 to 2008, respiratory samples from 520 patients were analyzed for the presence of HMPV by real-time RT-PCR. Molecular and phylogenetic analyses were performed on partial fusion gene sequences of HMPV-positive patients. *Results*. HMPV-positive patients were detected in 2007-2008. The overall infection rate was 4%, while 57% of the patients were children. Sequence analysis demonstrated circulation of subgroup B viruses with predominance of lineage B2. Nucleotide sequence identity within lineage B1 was 98.8%–99.7% and higher than that in lineage B2 (94.3%–100%). Three new amino acid substitutions (T223N, R229K, and D280N) of lineage B2 were observed. *Conclusion*. HMPV is a major viral pathogen in the Egyptian population especially in children. During 2007-2008, predominantly HMPV B2 circulated in Upper Egypt.

## 1. Introduction

*Human metapneumovirus* (HMPV) is an enveloped, single-stranded negative-sense RNA virus that has been classified in the *Metapneumovirus* genus of the Paramyxovirus family [1] and it is most closely related to respiratory syncytial virus (RSV) [2]. The genetic profile of the virus consists of 13,350 nucleotides, comprising the nucleoprotein (N), phosphoprotein (P), matrix protein (M), fusion protein (F), matrix proteins (M2-1 and M2-2), small hydrophobic protein (SH), glycoprotein (G), and RNA-dependent RNA polymerase (L) [3]. The fusion (F) and attachment (G) proteins are the major HMPV surface glycoproteins. The HMPV F gene codes for a class 1 viral fusion protein that mediates virus entry through both attachment [4] and fusion with the host cell membrane [5]. The HMPV F protein is a major antigenic determinant which mediates extensive cross-lineage neutralization and protection. Infection with each strain provided a high level of resistance to reinfection with the homologous or heterologous strain [6]. The F gene is relatively conserved between subgroups, making it an ideal target for nucleic acid amplification [7].

Evolutionary analyses suggest that it emerged from the closely related *Avian metapneumovirus C *(AMPV-C) approximately 200 years ago [8, 9]. Productive experimental infection of poultry with HMPV has not been successful, and serological studies have failed to detect evidence of human infection by AMPV [10]. Recent data suggest that the F protein is responsible for this species restriction. Thus, HMPV infection of humans may arise from a relatively recent transspecies transmission from AMPV-C [11].

There is a high seroprevalence of HMPV in sera of adults older than 40 years old, suggesting that it is a newly recognized rather than a newly emerging virus [10, 12–17].

Clinical symptoms caused by HMPV infection are similar to those of RSV and include fever, cough, wheezing, dyspnea, rhinorrhea, expectoration, and asthma [18–20]. Several studies have demonstrated that HMPV accounts for a high proportion of hospitalization for lower respiratory tract infections (LRTIs) in infants, young children, and other high-risk populations, such as immunocompromised patients [14, 21–23]. Rates of HMPV infection range from 5.5% to 25% among children hospitalized with respiratory illness [24–26].

The clinical manifestations of HMPV infection in adults depend on age and health conditions [2]. In young adults (aged 14–25 years), the clinical symptoms of HMPV infection are similar to those of other respiratory viral infections which include cough, rhinorrhea, and expectoration. In contrast, in middle aged (aged 26–65 years) and healthy elderly adults (aged > 65 years), HMPV is similar to influenza infections and common colds [2]. Although the incidence of HMPV infection in healthy adults during winter is generally less than 6% [27–29], HMPV is considered to be a major contributor to the burden of respiratory illnesses in older adults [29].

Phylogenetic analysis of HMPV has identified two subgroups, A and B. Both can be subdivided into their genetic lineages A1 and A2 and B1 and B2, respectively. All virus variants were identified in various countries in the Americas, Asia, and Europe [8, 30, 31].

Although reports have confirmed the prevalence of HMPV worldwide, data from North Africa and the Middle East remain limited. For the first time, the present study combines epidemiological data on HMPV prevalence and molecular, phylogenetic analyses of Egyptian HMPV.

## 2. Materials and Methods

### 2.1. Study Design

The study was conducted in Assiut University Hospitals. Assiut University Hospitals are one of the largest hospitals in Egypt and they predominantly give medical services to all the region of Upper Egypt. Upper Egypt is divided into nine governorates, which are Beni Suef, Menia, Assiut, Sohag, Qena, Luxor, Aswan, New Valley, and Red Sea.

From December 2005 to February 2008, a prospective study was conducted with patients admitted to the hospitals with community-acquired lower respiratory tract infection (LRTI). The study was approved by the medical ethical committee at Assiut University Hospitals, and oral consent was taken from the adult subjects or the children parents prior to sample collection.

LRTI was diagnosed clinically by the presence of cough as the main symptom, together with sputum production, dyspnea, wheeze, or chest discomfort/pain.

All participants were sampled at the same day or within 24 hours after admission to the hospital. Patients who were diagnosed with LRTI after 48 hours of admission (nosocomial LRTI) were excluded to ensure the diagnosis of community-acquired viral infections. During the period of examination, adults were enrolled during three consecutive winter-spring seasons, whereas children were enrolled in the season 2007-2008. According to Assiut University Hospitals' policy, children are those ≤14 years old, while adult patients were defined as patients > 14 years old. All children were presented at the pediatric outpatient clinic. Adults were either presented at the chest clinic or admitted to the chest department or the chest intensive care unit at Assiut University Hospitals (Table 1). Patients were asked to fill a questionnaire with demographic and clinical data including age, gender, residence, occupation, onset of symptoms, date and site of admission, clinical diagnosis, associated risk factors (smoking, associated cardiopulmonary condition, immunosuppressive condition, and other system affections), and antibiotic treatment.

### 2.2. Sample Collection

In total, 520 patients were prospectively enrolled in this study. To increase the number of virus-positive samples more than one specimen per patient was collected. Specimens were nasal swabs (NS), throat swabs (TS), nasal aspirates (NA), tracheal aspirates (TA), bronchoalveolar lavages (BL), gargles (G), and sputum (S). Samples were collected into sterile cups containing phosphate buffered saline as virus transport medium. Aliquots from each sample were done and stored at −80°C until the samples were finally shipped to the National Influenza Center, Robert Koch Institute, Germany, where the laboratory and the phylogenetic analyses were conducted.

### 2.3. RNA Extraction and Real-Time RT-PCR Assay

Viral genomic RNA was extracted from 400 µL of the original samples using RTP DNA/RNA Virus Mini Kit (Invitek, Germany) according to manufacturers' instructions. Real-time PCR assays were carried out to detect HMPV and other respiratory viruses including influenza A and B viruses [32, 33], respiratory syncytial virus (RSV A and B) [34], and adenovirus (AdV) [35]. A two-step RT-PCR method was used for the detection and amplification of HMPV fusion gene [36]. The reverse transcription step was performed with 25 µL of the viral RNA and 15 µL of a reaction mixture containing 200 µM deoxynucleoside triphosphates (dNTPs) (GE Healthcare, Austria), 5 mM dithiothreitol (DTT) (Invitrogen, Germany), 0.4 mM random primer (Invitrogen, Germany), 20-unit RNasin RNase inhibitor (Promega, USA), 100-unit Moloney murine leukemia virus reverse transcriptase enzyme (M-MLV) (Invitrogen, Germany), and a reaction buffer (Invitrogen, Germany). The reaction was carried out in the thermal cycler T3000 (Biometra, Germany) for 5 min at 42°C, followed by 30 min at 37°C and for 5 min at 94°C. Two sets of primer-probe pairs were used to detect and amplify the HMPV fusion (F) gene by real-time PCR (Table 2). The reaction mixture consisted of PCR buffer (Invitrogen, Germany), 100 µM dNTPs (GE Healthcare, Austria), 5 mM MgCl_2_ (Merck, Germany), 0.5-unit Platinum *Taq* DNA polymerase (Invitrogen, Germany), 500 nM of each primer (Tib Molbiol, Germany), 100 nM of each probe (Applied Biosystems, USA), and 3.0 µL cDNA with a final volume of 25 µL. The reaction was performed on the Stratagene Mx3000 and Mx3000P instruments. The PCR thermal profile consisted of an initial step of 5 min at 95°C, followed by 45 cycles each consisting of 15 s at 95°C and 30 s at 60°C.

### 2.4. PCR and Sequencing

Partial amplification of the F protein gene was performed for HMPV-positive samples by either external or seminested PCR. The reaction mixture was set up in a total volume of 50 µL containing PCR buffer, 100 µM dNTPs, 3 mM MgCl_2_ (Merck, Germany), 0.5-unit Platinum *Taq* DNA polymerase, 250 nM of each primer, and 5.0 µL DNA template. Amplification was done for 5 min at 94°C followed by 40 cycles at 94°C for 30 s, 60°C for 30 s, and 72°C for 45 s, with a final extension at 72°C for 10 min. Primers HMPV-3637-F, HMPV-4192-R1, and R2 were used for the first-round PCR (Table 2). The amplified products of 555 bp were visualized by GelRed or Ethidium bromide staining following electrophoresis on a 2% agarose gel. In case of negative results, 2 µL of the external PCR reaction was used for semi-nested PCR which was performed in a 50 µL reaction with 250 nM each of the primers HMPV-3637-F and HMPV-4164-R (Table 2). The nested amplicons of 527 bp were visualized by agarose gel electrophoresis as well.

Before sequencing, amplicons were purified either directly using the MSB Spin PCRapace kit (Invitek, Germany) or extracted from agarose gel using JETquick Gel Extraction Spin kit (Genomed, Germany) according to the manufacturer's instructions. Purified PCR products were cycle sequenced with primer pairs previously used for external and semi-nested PCR, respectively, in an ABI-Prism 3130xl Genetic Analyzer (Applied Biosystems, Germany) using the Big Dye Terminator v3.1 Cycle Sequencing kit (Applied Biosystems, Germany).

### 2.5. Phylogenetic and Molecular Analysis

A multiple sequence alignment was compiled from a part (439 nt) of the FI subunit of the F gene using ClustalW in the MEGA version 4.0 software program [37]. In MEGA, the Neighbor-Joining tree was calculated with the HKY85 model and a reliability of 1,000 replicates. The phylogenetic tree was rooted to *Avian metapneumovirus C* (AMPV-C). The following sequences of the described HMPV subgroups and lineages were included in the phylogenetic analysis: DQ009484 (AMPV-C), AY145288 (CAN98-73), AY145289 (CAN98-75), AY145290 (CAN98-76), AY145296 (CAN97-83), AY145297 (CAN00-12), AY145298 (CAN00-13), AY145299 (CAN00-14), AF371337 (NL/1/00), AY304360 (NL/17/00), Y304361 (NL/1/99), AY304362 (NL/1/94), DQ362939 (Arg/1/98), DQ362944 (Arg/1/99), DQ362947 (Arg/1/00), EU857567 (TN/94/1-1), EU857569 (TN/89/7-13), EU857570 (TN/97/2-37), EU857571 (TN/98/2-42), EU857572 (TN/87/2-7), EU857573 (TN/88/4-21), EU857581 (TN/99/4-19), EU857587 (TN/94/4-13), EU857592 (TN/92/10-32), EU857594 (TN/01/2-8), EU857595 (TN/98/5-12), EU857599 (TN/91/5-21), HQ456590 (GER/2499/04), HQ456598 (GER/0394/06), HQ456606 (GER/0513/07), HQ456611 (GER/0043/08), HQ456613 (GER/0259/08), HQ456617 (GER/0562/08), HQ456633 (GER/3379/10). The GenBank accession numbers of the Egyptian HMPV sequences are JQ041674, JQ041675, HQ909765, JQ041676, HQ909766, and JQ041677-JQ041695.

Pairwise nucleotide and amino acid identity within and between the genetic lineages B1 and B2 was calculated using the Bioedit version 7.2.3 [38].

### 2.6. Statistical Analysis

The SPSS program version 16.0 was used for the calculation of unpaired *t*-test and the Fisher's exact test, respectively.

## 3. Results

### 3.1. Study Population

From 2005 to 2008, a total of 520 patients with community-acquired LRTI were prospectively enrolled in this study. Almost all patients (99%) were residents of Upper Egypt and of different age groups (Table 1). Next to Egyptian patients, one patient was originally from Yemeni Republic who came to Egypt to seek medical care (Table 1). Children represented 13% (69 patients) of the study population, while adults represented 87% (451 patients). The mean age of children was 2.7 years and the median age was 1 year. For the adults, the mean age was 47.9 years and the median age was 49 years. Male children and adults constituted 55% and 61%, respectively. During the period of examination, samples from adults were collected within three consecutive winter-spring seasons, whereas children were prospectively enrolled in the season 2007-2008 (Table 3).

Of the 520 patients, 369 (71%) were hospitalized, 106 (20%) suffered from bronchopneumonia, 73 (14%) from acute bronchitis, and other furthers from different diseases of the lung (Table 1).

### 3.2. Detection of HMPV and Other Respiratory Viruses by Real-Time PCR

A viral agent was detected in 79 (15.2%) patients of the study population (Table 3). Thirty two of the positive patients were children (accounting for 46% of the total number of children enrolled in the study) and 47 were adults (accounting for 10% of the adult group). Of the positive patients, 22 (4.2%) were infected by influenza virus, 20 (3.8%) by adenovirus (AdV), and 16 (3%) by RSV. HMPV was detected in 21 patients (4.0%). In total, four coinfections were identified: three co-infections with RSV A and AdV and one coinfection with HMPV and AdV (Table 3).

**Figure 1:**
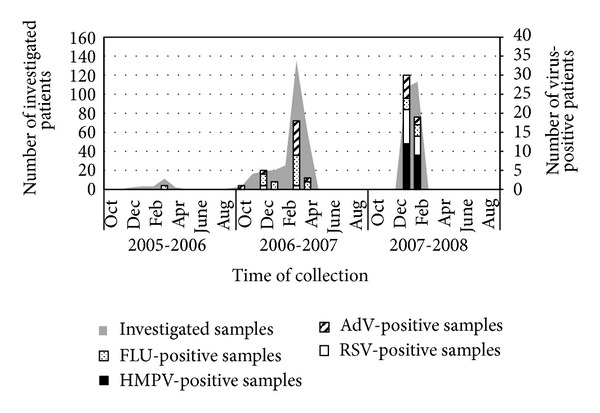
Monthly distribution of respiratory viruses causing LRTI. From 2005 to 2008, respiratory samples from patients with LRTI were collected and investigated for the presence of RSV, AdV, HMPV, and FLU. The gray shaded area in this figure shows the number of investigated samples per month. Each number of virus-positive samples is represented both with a bar and absolute values in the abscissa.

All HMPV-positive cases were detected during the season 2007-2008, while RSV and AdV were detected from 2006 to 2008. Influenza virus was present in all of the three seasons investigated (Table 3). The seasonal distribution of the respiratory viruses during the period of examination is shown in Figure 1. HMPV was detected in January and February of the season 2007-2008.

### 3.3. Characteristics of HMPV-Positive Patients

Among the twenty-one HMPV-positive patients, 12 (57%) were children. While all of the children were outpatients, 2 of 9 adults were inpatients admitted to the chest department. Thus, the majority (90%) of HMPV-infected patients were treated in ambulant medical surgeries (Table 4). Patients were primarily residents of Assiut (80%), followed by residents of the governarates Red Sea, Qena, Aswan, and Luxor (each 5%).

The most clinical signs significantly associated with the presence of HMPV were acute bronchitis (unpaired *t*-test, *P* = 0.022), bronchial asthma with infection exacerbation (*P* = 0.019), and bronchiolitis (*P* = 0.0001) (Table 4). Acute bronchitis was the most common diagnosis in adults (44%), whereas bronchiolitis was most frequently diagnosed in children (50%). Further, there was one two-year old patient (EG/347) coinfected with AdV. Patients EG/476 and EG/478 suffered from the underlying diseases bilateral hydronephrosis and hypertension, respectively.

**Figure 2:**
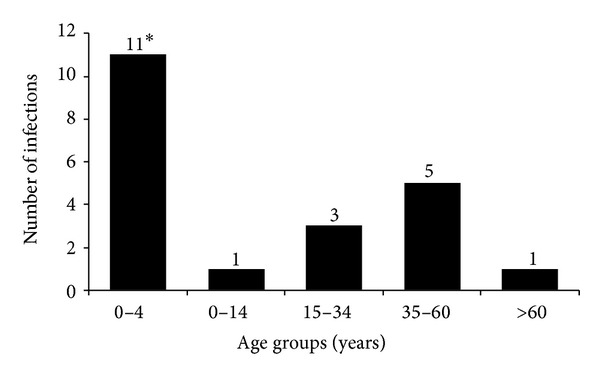
Number of HMPV infections in different age groups (*P = 0.0001).

To describe the circulation of HMPV in the Egyptian population, patients were grouped by age: 0–4 years, 5–14 years, 15–34 years, 35–60 years, and >60 years (Figure 2). HMPV infections occurred in patients from all age groups but were most common among children between 0 and 4 years (Fisher's exact test, *P* = 0.0001).

### 3.4. Phylogenetic Analysis of HMPV

**Figure 3:**
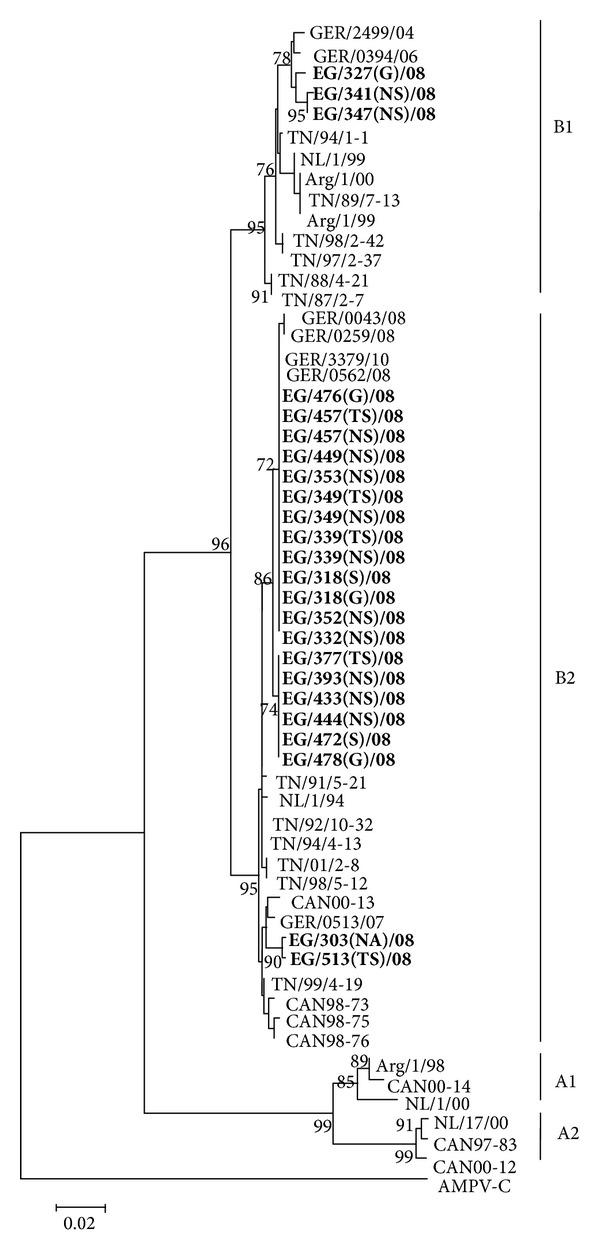
Phylogenetic tree of partial F gene sequences of Egyptian HMPV. The tree was generated with Neighbor-Joining method with 1,000 bootstrap replicates and rooted to *Avian metapneumovirus C *(AMPV-C). Reference sequences representing the different HMPV genetic lineages were additionally included in the analysis. Egyptian viruses are shown in boldface. These viruses are identified by the geographic location, patient number, type of specimen, and year of isolation. The scale bar represents 2% of nucleotide changes between close relatives.

Partial amplification of the F protein gene was performed for 32 specimens of the 21 HMPV-positive patients. Except for patient EG/459, partial fusion gene sequences were obtained for all other patients. Sequences were obtained from 24 specimens out of the 32 HMPV-positive specimens (75%). Phylogenetic analysis was conducted on Egyptian HMPV sequences of 439 nt of the F gene. At present, HMPV is differentiated into subgroups A and B, each with two genetic lineages A1, A2, B1, and B2, respectively. All the Egyptian HMPV sequences clustered in subgroup B (Figure 3). Of the 24 Egyptian sequences 3 belonged to lineage B1 and 21 to lineage B2. Thus, 3 patients (15%) were infected with HMPV of lineage B1 and 17 patients (85%) were infected with HMPV of the lineage B2. There were Egyptian HMPV sequences identical (GER/3379/10, GER/0562/08) or closely related (GER/0394/06, GER/0259/08, and GER/0043/08) to sequences from Germany. Further, the Egyptian sequences clustered in batches of nearly identical (lineage B1) or identical (in lineage B2) sequences. HMPV sequences EG/303(NA)/08 and EG/513(TS)/08 of lineage B2 clustered separately from the other Egyptian sequences of the same lineage. These sequences were detected in patients resident in Red Sea and Luxor.

### 3.5. Molecular Analysis of HMPV

The nucleotide identity between lineages B1 and B2 was 94.3%–95.2%, whereas it was 98.8%–99.7% and 94.3%–100% within lineages B1 and B2, respectively (Table 5). The sequences between lineages B1 and B2 shared an amino acid identity of 97.2%–98.6% and within lineages B1 and B2 of 99.3%–100% and 97.2%–100%, respectively. Lineage B2 was the most divergent. The analysis of sequence similarity showed greater nucleotide than amino acid diversity as determined by other studies [8, 18, 39] from for example, The Netherlands, Canada, or USA, respectively (Table 5).

**Figure 4:**
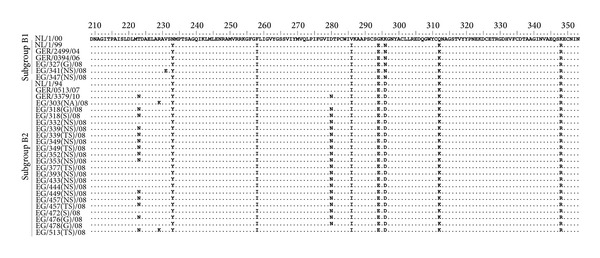
Amino acid alignment of partial F protein gene of HMPV. Predicted amino acid sequences of Egyptian HMPV B were compared with the corresponding sequence of the prototype strain NL/1/00 (GenBank accession number AF371337). Identical residues are indicated by dots. The Egyptian HMPV is identified by the geographic location (EG), patient number, type of specimen, and year of isolation.

There were five amino acid residues (233, 286, 296, 312, and 348) common to subgroup B strains (Table 6). Single amino acid variations were present in one, two, or few sequences. The highest amino acid variability was observed for twelve sequences of the lineage B2 (Figure 4). They all exhibit additional unique amino acid substitutions at residue 223 and 280. Moreover, phylogenetic analysis (Figure 3) revealed that these twelve sequences clustered separately and were closely related to sequences from Germany (GER/3379/10 and GER/0513/07). Interestingly, there were two Egyptian strains (EG/303(NA)/08 and EG/513(TS)/08) which comprise an additional but different amino acid substitution at residue 229 (Table 6). These sequences clustered separately from the other Egyptian strains (see above).

## 4. Discussion

This study was performed to estimate the prevalence of HMPV in patients with community-acquired lower respiratory tract infection in Upper Egypt, and more important, to give new insights into circulating HMPV strains in Egypt at all.

The overall proportion of HMPV infections in Upper Egypt between 2005 and 2008 was 4% with a frequency rate of 17% in the children's group. In the area of greater Cairo (Egypt) the positive rate in children was 6.4% in 2006-2007 [40]. Another study of HMPV infection in children of Mansoura, northeast part of Egypt, in 2010 showed a positive rate of 8% [41]. Further, in comparison to our study, the infection rate of HMPV in children is at an equal frequency level in the Arabian Peninsula. From July 2007 to November 2008 an incidence of 8.3% was observed in Saudi Arabia [42], whereas the frequency rate of HMPV was 12.7% in hospitalized children from Jordan in 2007 [43].

While the HMPV frequency in children was 17%, the infection rate in the adult population was 2% in our study. In a different study from Egypt from 2007 to 2009, a positive rate of 13.6% was observed [19], which is higher than the infection rate of adults in this study. The higher infection rate of HMPV in children supports the hypothesis that HMPV is a leading cause of respiratory tract infection in the first years of life [44]. In general, the HMPV frequency rate varies worldwide from 3.5% in Finland to 17.8% in Brazil [45–50]. Altogether, the frequency of HMPV varies from year to year in a given region depending on the geographical locations, and furthermore the positive rate alters due to different methods of sample collection and variation of calculation methods [43, 51].

The clinical manifestations of HMPV-infected patients are mainly mild upper-airway diseases [52]. In our study, this is reflected by the presentation of HMPV-infected patients at the outpatient clinic (90%). Nevertheless, HMPV can be associated with severe disease of the lower respiratory tract. One major clinical diagnosis in the present study was pneumonia which was found in 20% of the infected HMPV patients. Similar findings were observed in Egyptian adults in the same time period (2007–2009) with a frequency of 17% for pneumonia [19]. The common clinical diagnosis noted in children with HMPV in the present study was bronchiolitis, which can be supported by data from Egypt, USA, and Brazil [23, 41, 53].

Next to single HMPV infection, co-infections of HMPV with different respiratory pathogens occur. The co-infection rate (0.8%) in this study was low. We detected three co-infections in children (4.3%) and one co-infection in adults (0.2%). With regard to HMPV, there was one co-infection with HMPV and AdV identified in an infant. In a different study from Egypt conducted in 2006-2007, a comparable co-infection rate in children (4.5%) to our results was observed [40]. Interestingly, a study in China revealed a co-infection frequency of 58% in HMPV-positive children [54]. Co-infections with HMPV are often described for children [55, 56] and were mostly associated with the age group less than 6 months old [57].

To investigate the genetic variability of the HMPV strains in Upper Egypt, a stretch of 439 nt of the fusion gene was analyzed. The phylogenetic analysis was both performed with German strains and other published reference strains representing HMPV subgroups A and B. The Egyptian sequences clustered in HMPV subgroup B with predominance of the genetic lineage B2. Whereas in Upper Egypt HMPV B circulated in the season 2007-2008, in Jordan (Arabian Peninsula) both subgroups were detected in 2007 [43]. HMPV subgroup A was detected in 93% of the patients and 28.6% of the patients were infected with subgroup B. Co-infection with HMPV subgroups A and B was detected in 21,4% [43]. These different circulation patterns in the same area support the assumption that the circulation of HMPV subgroups and sub-clusters might be a local phenomenon [58].

In this study, a high degree of identity was observed in the F gene among most of the Egyptian sequences at both the nucleotide and amino acid levels. In line with these findings, the F gene was well conserved in HMPV circulating in Europe, North America, and Asia [8, 18, 31, 59–61] (Table 5). The amino acid conservation of the F gene was higher in the Egyptian strains than nucleotide sequence conservation. This is supported by the assumption that nucleotide diversity is greater than amino acid diversity among HMPV F sequences due to functional constraints on fusion proteins which prevent drastic amino acid changes. This could lead to a lack of directional antigenic drift in paramyxoviruses in contrast to influenza viruses [62].

Our phylogenetic analysis demonstrated the cocirculation of the genetic lineages B1 and B2 during season 2007-2008. HMPV lineage B2 was predominant in that season. The Egyptian HMPV strains formed three clusters, two in the B2 branch and one in the B1 branch. In regard of the amino acid substitution, it was revealed that B2 strains were more variable than B1 strains. Two Egyptian HMPV (EG/303(NA)/08 and EG/513(TS)/08) showed a unique amino acid substitution in the F gene resulting in an allocation of these strains to a separate branch from B2 viruses. These viruses were detected in two patients from Red Sea and Luxor. Interestingly, there were Egyptian and German HMPV strains which were very closely related. The relation of HMPV from distant regions is known but reasons remain unclear. It can be supposed that human travelling behaviour influences the diversity and evolution of HMPV.

## 5. Conclusion

*Human metapneumovirus* is an important respiratory viral pathogen in the Egyptian patient population especially children. The phylogenetic analysis of Egyptian HMPV isolates showed the circulation of viral group B during season 2007-2008 with predominance of lineage B2. Our study provides new insights into the epidemiology of HMPV in Egypt.

## Figures and Tables

**Table 1:** Demographic and clinical characteristics of patients.

Patients' characteristics	*N* (%)
Age in years (*n* = 520)	
0–14	69 (13)
15–34	88 (17)
35–60	275 (53)
>60	88 (17)
Sex	
Girls	31 (45)
Boys	38 (55)
Female	178 (39)
Male	273 (61)
Geographical area	
Upper Egypt	
Bani Suif	6
Menia	27
Assiut	307
Sohag	62
Qena	47
Luxor	18
Aswan	32
New Valley	9
Red Sea	7
Lower Egypt	
Suez	3
Alexandria	1
Yemeni Republic	
Yemeni Republic	1
Site of admission	
Pediatric outpatient clinic	69 (13)
Chest outpatient clinic	82 (16)
Chest department (inpatient)	264 (51)
Chest intensive care unit (inpatient)	105 (20)
Clinical diagnosis	
Bronchopneumonia	106 (20)
Acute bronchitis	73 (14)
Bronchial asthma with infectious exacerbation	72 (14)
Bronchiolitis	29 (6)
Lobar pneumonia	28 (5)

**Table 2:** HMPV oligonucleotide primers and probes.

Name	Oligonucleotide sequence (5′-3′)	Position^a^	Gene	Polarity	Reference
Real-time PCR					
HMPV F S	gCTCCgTAATYTACATggTgCA	794–815	F	+	[36]
HMPV F S1	gAAgCTCYgTgATTTACATggTYCA	791–815	F	+	[36]
HMPV F AS	gACCCTgCARTCTgACAATACCA	924–947	F	−	[36]
HMPV F AS1	AgTKgATCCTgCATTTTTACAATACCA	924–951	F	−	[36]
HMPV F TMGB	F-CCYTgCTggATAgTAAAA-MGB	844–861	F	+	[36]
HMPV F TMGB1	F-CCTTgTTggATAATCAA-MGB	844–860	F	+	[36]

Conventional PCR and sequencing					
External PCR					
HMPV-3637-F	gTYAgCTTCAgTCAATTCAACAgAAg	571–596	F	+	[31]
HMPV-4192-R1	CAgTgCAACCATACTgATRggATg	1101–1125	F	−	[36]
HMPV-4192-R2	TAgTgCAACCATACTgATRgggTg	1101–1125	F	−	[36]
Seminested PCR					
HMPV-3637-F	gTYAgCTTCAgTCAATTCAACAgAAg	571–596	F	+	[31]
HMPV-4164-R	CCTgTgCTRACTTTgCATggg	1077–1097	F	−	[36]

^a^Nucleotide positions are given according to the gene positions in HMPV isolate NL/1/94 (GenBank accession number AY304362). The base “G” is given in lower letters to avoid confusion with “C.” Abbreviations: F: 6′-carboxyfluorescein (FAM); MGB: minor groove binder; nucleic acid codes: Y: C/T, R: A/G, and K: T/G.

**Table 3:** Detection of respiratory viruses in patients with LRTI (children/adults).

Season	Number of investigated patients	Virus-positive patients				
		HMPV	RSV	Influenza virus	Adenovirus	Number of coinfections
2005-2006	22 (0/22)	0	0	1	0	0
2006-2007	278 (0/278)	0	2	15	12	0
2007-2008	220 (70/150)	21 (12/9)	14 (12/2)	6 (1/5)	8 (7/1)	4 (3/1)

Total	520 (69/451)	21 (12/9)	16 (12/4)	22 (1/21)	20 (7/13)	4 (3/1)
Percent	100 (13/87)	4.0 (17/2)	3 (17/1)	4.2 (1/5)	3.8 (10/3)	0.8 (4/0)

**Table 4:** Clinical characteristics of HMPV infections.

Patient	Age	Gender	Residence	Smoking pattern	Specimens	In-/outpatient	Underlying disease	Clinical diagnosis
EG/303	56 y	F	Red Sea	None	NA, G, and S	Outpatient	None	Acute bronchitis
EG/318	27 y	F	Qena	None	NS, G, and S	Outpatient	None	Acute bronchitis
EG/327	55 y	M	Assiut	None	NS, G, and S	Outpatient	None	Bronchial asthma with infectious exacerbation
EG/332	6 mo	M	Assiut	None	NS, TS	Outpatient	None	Bronchiolitis
EG/339	2 y	M	Assiut	None	NS, TS	Outpatient	None	Bronchiolitis
EG/341	2 y	F	Assiut	None	NS, TS	Outpatient	None	Bronchiolitis
EG/347	2 mo	F	Assiut	None	NS, TS	Outpatient	None	Bronchiolitis
EG/349	2 y	F	Assiut	None	NS, TS	Outpatient	None	Bronchial asthma with infectious exacerbation
EG/352	2 y	M	Assiut	None	NS, TS	Outpatient	None	Bronchiolitis
EG/353	6 mo	F	Assiut	None	NS, TS	Outpatient	None	Bronchiolitis
EG/377	3 y	F	Assiut	None	NS, TS	Outpatient	None	Bronchial asthma with infectious exacerbation, tonsillitis, and pharyngitis
EG/393	2 y	M	Assiut	None	NS, TS	Outpatient	None	Bronchial asthma with infectious exacerbation, tonsillitis, and pharyngitis
EG/433	24 y	M	Assiut	Mild	NS, TS, and G	Inpatient	None	Multiple pyemic abscesses (septic embolism), acute infective septic endocarditis
EG/444	22 y	F	Assiut	None	NS, G	Outpatient	None	Lobar pneumonia
EG/449	1 y	M	Assiut	None	NS, TS	Outpatient	None	Bronchopneumonia
EG/457	8 mo	M	Aswan	None	NS, TS	Outpatient	None	Bronchopneumonia
EG/459	5 y	F	Assiut	None	NS, TS	Outpatient	None	Acute bronchitis, tonsillitis, and pharyngitis
EG/472	60 y	F	Assiut	None	NS, G, and S	Outpatient	None	Acute bronchitis
EG/476	47 y	M	Assiut	None	NS, G	Outpatient	Bilateral hydronephrosis	Acute bronchitis
EG/478	56 y	F	Assiut	None	NS, G	Outpatient	Hypertension	Bronchial asthma with infectious exacerbation
EG/513	66 y	M	Luxor	None	NS, TS, and BL	Inpatient	None	Bronchopneumonia

Abbreviations: NS: nasal swab; G: gargle; TS: throat swab; BL: bronchoalveolar lavage; S: sputum.

**Table 5:** Genetic similarity between and within HMPV subgroups and lineages (F, fusion gene).

Study	Country	Nucleotide sequence identity (%)				Amino acid sequence identity (%)			
		B1	B2	B1-B2	A-B	B1	B2	B1-B2	A-B
**This study**	**Egypt**	**98.8–99.7**	**94.3–100**	**94.3–95.2**	**—**	**99.3–100**	**97.2–100**	**97.2–98.6**	**—**
[8]	The Netherlands	97–100	97–100	94–96	84–86	99-100	99-100	97–99	94–97
[18]	Canada	—	—	94.3–99.9	83.0–83.6	—	—	98.3–99.8	94.1–95.4
[31]	Germany	97.1–99.8	98.3–99.5	94.0–95.7	83.6–87.4	—	—	—	—
[59]	Many countries	96.0–99.9	97.2–99.4	92.0–94.1	81.5–85.3	99.5–100	99.1–100	98.1–99.1	93.1–96.3
[39]	USA	98–100	96–100	93–95	—	100	99.4	98.4	—
[60]	France	97.2–100	97.2–100	92.6–94.4	82.7–86.5	—	—	—	—
[61]	Cambodia	—	97.3–100	92.6–100	—	—	97.8–100	97.2–100	—

**Table 6:** Lineage-specific amino acid substitutions in the HMPV F gene between positions 209 and 353.

Lineage	Amino acid position in the F gene of Egyptian sequences								
	223	229	231	233	280	286	296	312	348
B1 (*n* = 3)		R→K (*n* = 2)	V→E (*n* = 1)	Y		I	N	K	R
B2 (*n* = 21)	T→N (*n* = 13)			Y	D→N (*n* = 19)	I	D	K	R
